# The Effects of Using a Low-Cost and Easily Accessible Exercise Toolkit Incorporated to the Governmental Health Program on Community-Dwelling Older Adults: A Quasi-Experimental Study

**DOI:** 10.3390/ijerph19159614

**Published:** 2022-08-04

**Authors:** Shih-Hsien Yang, Qi-Xing Chang, Chung-Chao Liang, Jia-Ching Chen

**Affiliations:** 1 Department of Rehabilitation Medicine, Hualien Tzu Chi Hospital, Buddhist Tzu Chi Medical Foundation, Hualien 970, Taiwan; 2 School of Medicine, College of Medicine, Tzu Chi University, Hualien 970, Taiwan; 3 Institute of Brain Science, National Yang Ming Chiao Tung University, Taipei 112, Taiwan; 4 Department of Physical Therapy, College of Medicine, Tzu Chi University, Hualien 970, Taiwan

**Keywords:** health promotion, community care station, older adults, physical performances

## Abstract

The Community Care Station (CCS) service was initiated by the Taiwanese government as a part of its elderly social services programs. This study aimed to investigate the effects of using an inexpensive exercise toolkit, containing a stick, theraband, sandbag and a small ball, led by a physical therapist among community-dwelling older adults participating in CCS. A total of 90 participants (aged 77.0 ± 6.8 years) were recruited and divided into an intervention group (*n* = 45) and a comparison group (*n* = 45). The intervention group regularly participated in a health promotion program with the exercise toolkit for approximately 90 min per twice-weekly session for 3 months, and the comparison group maintained their usual CCS activity program. Both groups were assessed before and after the 3-month intervention period. Outcome measures included the Short Physical Performance Battery (SPPB), one-leg stance, functional reach (FR), Timed Up and Go (TUG), and 10 m walk tests; 83 participants completed the study. No significant between-group differences were found at baseline in general characteristics or outcome variables. After 3 months, the intervention group showed the significant group x time interaction effects in SPPB, one-leg stance, FR, TUG and 10 m walk tests compared to the comparison group (*p* < 0.05).; A structured group-based health promotion program using a low-cost exercise toolkit could be effective in improving the physical performances, balance, and walking ability of community-dwelling older adults receiving CCS program services. Furthermore, the comparison group maintained most of their physical performances, even showing significant progress on FR.

## 1. Introduction

Toward the end of the 20th century, aging societies became a global public health and social issue, especially in advanced countries [1]. In view of the ratio of aging population and the aging rate, Taiwan has been one of these aged countries and the population aging speed is one of the fastest worldwide, predicting toward 20% by 2025 and becoming the super-aged society, which make this phenomenon become a public health priority [2].

Evidence has shown that physical performance and strength decline with age in healthy people [3,4,5,6,7,8,9,10,11]. Furthermore, older adults either with self-irregular exercise, low physical activity level or a sedentary lifestyle could regress in their physical performance and strength with age, which is strongly related to increased chances for frailty, the risks of falling, disability, institutionalization and mortality [7,8,9,10,11,12]. Therefore, to delay the age-related declination of strength and physical mobility and prevent the disability among the community’s older adults, official or non-official institutions in these countries have developed various community-based service and health promotion programs, such as Enhance^®^ Fitness program, Feeling Fit Club in the USA [13,14], community-based integrated care in Japan [15,16], and community care center (station) in Taiwan [17,18,19,20]. However, owing to the diverse culture and lifestyle of older populations, different social welfare and healthcare policies, and the contents of these health programs among these various countries [13,14,15,16,17,18,19,20,21,22,23], there could be no “one size fits all” to deal with the “aging” problem.

The Community Care Station (CCS) service is one of the most prominent strategies under the “Taiwan Healthy Community 6-Star Plan,” which was a public health and senior welfare policy launched in 2005 by the Taiwanese government in response to the rapid and massive aging society [13,14,15,16,17,18,19,20]. By using unused local community centers, churches, temples, etc., CCSs provide social and eldercare services for community older adults. The contents of CCS service program have been described elsewhere [18,20]. According to the update estimation from the Taiwanese government, the service, initiated from 300 locations, has expanded to approximately 4000 locations as of 2020 [18], indicating the service model is apparently popular and well-accepted by older adults in Taiwanese communities. Although the CCS service model has developed in Taiwan for many years, studies reported that the effectiveness of the governmental health programs with incorporating physical activity (PA) and exercise to investigate the physical function and strength among the community older adults is still growing and needs to be validated [21].

Concerning the previous studies conducted in the CCS service model or community care center (station) in Taiwan, they were either specific to a single component, such as only using theraband for muscle strengthening, or focused on a certain type of physical activity and exercise investigation, such as yoga, acupunch, or even only emphasized a health education course without doing exercise for the community older adults [19,20,24,25,26,27]. In addition, parts of them were conducted with a single-group study design [19,20,24], and diverse outcome measures and various program contents used among these studies made consistent results difficult [19,20,21,22,23,24,25,26,27]. Another, according to the American College of Sports Medicine (ACSM) and systematic review recommendations, the multicomponent programs involving diverse physical capacities, such as flexibility, strengthening, balance and endurance, are more effective for the physical performance and healthy status of the community older adults [28,29,30]. From a practical, public health, cost-effectiveness and long-lasting perspective under the government-promoted health program, it could be valuable and critical to design an exercise protocol including domains of flexibility, strength and balance by using a package of low-cost and accessible exercise tools for the community older adults participating in CCS services, and investigate its effects on physical mobility and function.

Therefore, the purpose of this study was to investigate a structure group-based exercise by using a low-cost modality including a stick, theraband, sandbag and a small ball, as packaged in an exercise toolkit, applied in a CCS for 3 months, and compared with a comparison group without using this set of exercise aids, to understand the effects on the physical performance of community-dwelling older adults after participating in CCS services.

## 2. Materials and Methods

The study was conducted at north area of Hualien County in Taiwan. A total of eight CCSs due to proximity to the researchers of this study were invited to the research plan by visiting the center administrators and participants. Finally, 90 participants were recruited from four CCSs and volunteered to participate. By convenience sampling, all of them were assigned to the intervention group for two CCSs (*n* = 45) and the comparison group for two CCSs (*n* = 45) based on the CCSs in which they participated. All the community older adults participating in the study met the following criteria: (1) age ≥ 65 years and living in the community; (2) less than 3 errors in the Short Portable Mental State Questionnaire (SPMSQ) [31]; and (3) able to walk independently with or without a device. The exclusion criteria were: (1) psychiatric illness, epilepsy or related medical history; (2) unstable medical conditions such as angina pectoris and acute myocardial infarction; and (3) receiving rehabilitation care (e.g., outpatient rehabilitation service) or practicing other physical activities such as Tai Chi during the study. All participants received and signed the consent form approved by the Research Ethics Committee of Hualien Tzu Chi Hospital, Buddhist Tzu Chi Medical Foundation, registered as number IRB-106-45-A, and the study complies with the Declaration of Helsinki.

### 2.1. Intervention

All participants in both groups regularly participated in CCS services (3 h, twice-weekly sessions). In the intervention group, each of the participants was given an exercise toolkit containing a stick (length 100–110 cm) or trekking pole for substitution, theraband, sandbag and a small ball led by a physical therapist for 2 sessions a week, taking place for 3 consecutive months. The 3 h CCS sessions included 90 min of multicomponent physical exercise sessions designed to improve flexibility, strength, balance and endurance.

The exercise program consisted of three phases. During implementation, all older adults were arranged into a “U” shape and a therapist, located in the middle, demonstrated the exercise and asked participants to follow. The first phase consisted of range-of-motion exercises on all extremities using a stick and also combining trunk mobility exercises with stretching and flexibility for 20 min, including 5–10 min for warm-up.

**Figure 1:**
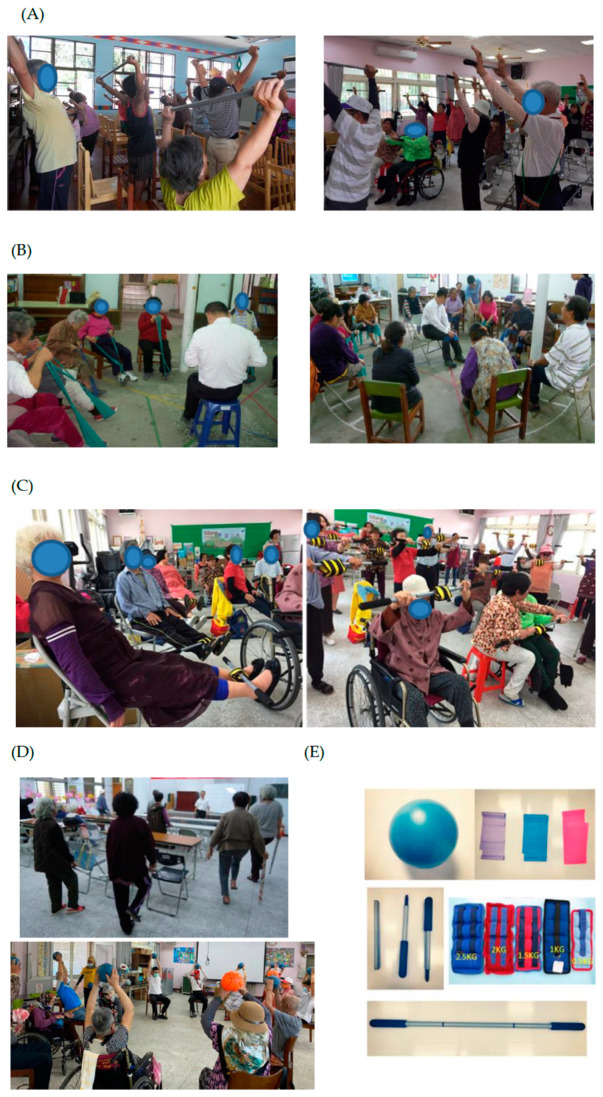
The exercise program by using exercise toolkit in three phases for the community older adults participating in the CCS program. (**A**) Stretching and flexibility exercises with a stick or trekking pole. (**B**) Resistance training for upper and lower limbs by using a theraband. (**C**) Using a stick combing with a sandbag for muscle strengthening for upper and lower limbs. (**D**) Balance training with or without support by chair or stick and coordination by using a small ball. (**E**) The exercise toolkit containing a stick (length 100–110 cm) or trekking pole, therabands, sandbags and a small ball.

In the second phase, muscle strengthening was performed for approximately 30 min to train 4–5 muscle groups in the upper limb and 5–6 muscle groups in the lower limb, interleaving 5–10 min between upper and lower limbs strengthening. Each muscle group exercise initially comprised 2–3 sets with 6–8 repetitions each, using sandbags or differently colored therabands (representing different resistances). Resistance intensity progressed following monthly reevaluations, finally aiming for 3 sets of 10–12 repetitions for each muscle group. The sandbag and theraband were alternatively used as training tools due to providing different types of muscle strengthening. Both tools would also sometimes be separately tied on the stick to provide various muscle strengthening techniques. In muscle strengthening with a sandbag, the weight would be modulated starting from 0.5–1 kg, then incremented by 0.5 or 1 kg each time depending on the individual’s tolerance and performance, toward a maximal 2 kg for women and 2.5 kg for men following monthly reevaluation by the therapist until the end of the study. The therabands also went from yellow and blue to green and black (i.e., from low to high resistance intensity). During the training period, community volunteers assisted the older adults with lesser performance by supervising the exercise. The participants were allowed to decrease the number of repetitions or not to adjust their resistance (i.e., maintaining their sandbag weight and theraband color) if they felt beyond their tolerance. In the third session, the therapist turned to performing balance and endurance exercises using the items in the Berg balance scale [32], from static to dynamic activity, and sitting to standing, and alternating stepping for 20 min, finally cool-down for 5–10 min [29]. The therapist would also modify these balance exercise items depending on participants’ performance, such as from close feet to semi-tandem to tandem position and with support to without support from a stick or chair. The physical exertion intensity of the older adults was modulated no more than 11–13 points on the Borg Rate of Perceived Exertion (RPE) scale [33]. The exercise program by using exercise toolkit in three phases for the participants in the intervention group is presented in Figure 1.

In the comparison group, the older adults participated in the same number of CCS sessions and only received the usual program without the exercise package and toolkits (i.e., sessions were run by local volunteers). The usual program in these sessions comprised computer games, handicraft, playing chess, singing or painting, video watching, health education, etc. for nearly one hour. Only two of the above-mentioned items were scheduled in each session. During the second hour, general physical activities, such as a range-of-motion exercises for upper and lower limbs and self-stretching exercises for 10–15 min were followed by physical or recreational activities such as throwing and catching, kicking differently sized balls lasting for approximately 30–40 min in each session. Participants in both groups were provided with a hot meal for lunch after total activity course end, lasting approximately 30–40 min. During this phase, these participants usually chatted with each other.

For consistency, community volunteers had to receive two 8 h training sessions about the activity and care courses conducted in the CCSs, disseminated by volunteers before the study began. The training courses were held by the local government and assisted by a physical therapist not involved in the study and with 10-year community service program experience.

### 2.2. Assessments

The Short Physical Performance Battery (SPPB) is frequently used as a physical performance test for older adults and involves 3 item tests. Each item is scored 0 to 4 points, giving a total score range of 0–12 points; higher scores mean better physical mobility [34]. The one-leg stance (OLS) test is a clinical tool to assess postural steadiness while standing. The participant was asked to “flex one knee and stand on the other leg (your dominant leg) as long as possible.” Two standing times were recorded, and the times were averaged [35,36]. Functional reach (FR), which measures the distance between the length of the middle-fingers’ tips and a maximal forward reach while maintaining 90° shoulder flexion and straight arms in the standing position, can be used to evaluate the balance ability of older adults. The mean value of 2 repetitions was recorded as their performance [36]. The Timed Up and Go (TUG) test is a common measure to determine dynamic balance. The older adults were timed when they rose from a chair, walked 3 m at a comfortable and safe pace, turned around, walked back to the chair, and sat down again. The average time of 2 attempts was recorded [36,37]. The 10 m walk test assesses the walking ability of older adults. Older adults walked at their usual walking speed for 5 m each of acceleration/deceleration, and the time needed to walk to the midpoint of 10 m was recorded. The average time of 2 attempts was used for analysis [38]. We summarize all the outcome measures, their objective, conditional capacity or skill evaluated, and mean reference values in Table 1.

All participating community older adults were screened and evaluated by physicians before the tests, and one physical therapist who was independent of the study conducted all assessments before and after the study.

### 2.3. Statistical Analysis

Although the present study was not randomized, we still calculated the sample size and required 72 participants for the effect size 0.6, as by G-power statistic with power 0.8, alpha 0.05, with a mean difference between the two groups of 1.2 points (SD = 2.0 points) in SPPB [34]. The demographic data and performance tests before intervention in both groups were compared using the χ^2^ test for categorical variables or the independent *t* test. The difference in all physical outcomes before and after intervention was analyzed by 2-way repeated measures analysis of variance (ANOVA) within and between groups. Bonferroni correction was used for post hoc analysis. Statistical analysis was performed using SPSS 24.0 and the significant difference *p* value was set at < 0.05.

## 3. Results

At the end of the study, a total of 83 (42 from the intervention group and 41 from the comparison group) completed pre/post assessments. There were 7 dropout participants in the study (4 from the comparison and 3 from the intervention group), who moved away (*n* = 3), died (*n* = 1), were admitted to the hospital (*n* = 2) or left for unknown reasons (*n* = 1). No adverse event was reported by the participants during the study period. Attendance rates were 91% in the intervention group and 88% in the comparison group (*p* > 0.05), respectively. The common reasons for the participants who missed their CCS program classes were personal factors, having a trip and visiting their relatives or friends in other places.

**Figure 2:**
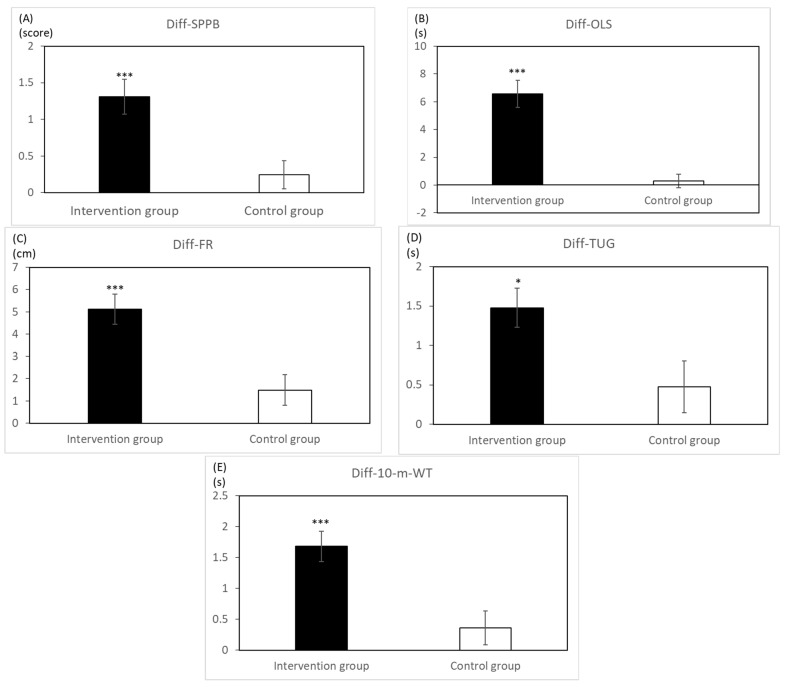
Demonstration of post−pre difference in five mobility performance of the intervention (black bar) and control (white bar) groups. (**A**) Diff−SPPB, the mean score of SPPB at post test−the mean score of SPPB at pretest. (**B**) Diff−OLS, the mean score of OLS at post test−the mean score of OLS at pretest. (**C**) Diff−FR, the mean score of FR at post test−the mean score of FR at pretest. (**D**) Diff−TUG, the mean score of TUG at pretest−the mean score of TUG at post test.; (**E**) Diff−10−m WT, the mean score of 10−m WT at pretest−the mean score of 10−m WT at post test. Statistics by two way repeated measures ANOVA. *: *p* < 0.05; ***: *p* < 0.001.

Table 2 shows the basic characteristics and epidemiological data of all older adults in both groups. No significant differences at baseline were found between the two groups’ basic characteristics. After the 3-month intervention, the intervention group showed significant time effects in all physical outcomes, and the significant interaction (group by time) effects compared with the comparison group shown in Table 3. The only significant improvement in the comparison group after the intervention was in FR (*p* < 0.05). Figure 2 presents the within-group mean change in all physical performance tests between the 2 groups and demonstrates significant gains of most physical performance in the intervention group as compared with the comparison group.

## 4. Discussion

The present study demonstrated that a 3-month structured group-based exercise, by using an inexpensive and accessible exercise toolkit (less than NT 600 in a set of exercise toolkit) incorporating to the CCS service model, could improve physical performance among community-dwelling older adults compared to the comparison group. The study also showed the comparison group maintained most of their mobility performance, and even made significant progress on FR by means of assistance from local community volunteers.

The following reasons could explain why the type of half-day activity program with multi-components in this study could do many characteristics for the community older adults. First, the CCS services can provide social support and contact for these older people, especially for the isolated or live-alone person. Because of living in the same community or nearby, they are either neighbors or know one another. Therefore, for “the day of physical activity course”, they can go together to the station to participate in this type of physical activity and exercise. Second, owing to funding by the local government or non-official organization, physical activity classes are free or low-cost. These community older adults not only attended the classes but had their lunch in each session in the station before going home [14,18,20,22]. In view of the isotemporal substitution model [12], the participants reallocated their time spent in physical activity and exercise instead of staying at home or leading a sedentary behavior in half-day. Third, the community center is accessible and convenient for these older adults without shuttle bus transportation. Therefore, they can not only walk or ride the mobility scooters to CCS but gain a sense of belonging and enjoyment, and establish friendships [40], which could motivate them to continue with the activity and result in high adherence in this study. Fourth, when they performed the health-promotion exercise program, they had good rapport with the therapist, with appreciated friendly and fair competition [40]. Finally, and most importantly, the exercise volume and intensity could be more in the intervention group than the comparative group. As a dose-response relationship [41], the participants in the intervention group performed nearly 90 min of multi-component exercise, including flexibility, resistance and balance, contrasting to throwing and kicking ball(s) or the recreational activity in the comparative group.

Compared to these previous studies, the health promotion exercise program adopted in the present study might not be novel. Nevertheless, the structured package designed by the physical therapist (using a stick for stretching and flexibility initially, then resistance muscle strengthening of upper and lower extremities using therabands and sandbags, and finally incorporating balance and coordination with or without the stick and a ball) could be the first to be implemented among these participants in the community setting. All of the exercise tools in this structured package cost approximately USD 20 a set, under instruction by a physical therapist are easily accessible, convenient, inexpensive and without a space to setting. The training components also match the exercise elements for the elderly recommended by ACSM [29]. As a result, we observed significant improvement on all physical tests in the intervention group. Furthermore, we have also found the SPPB score and gait speed in the intervention group improved 1.3 points vs. 0.17 m/s from pretest to post test, respectively, which showed the magnitude of improvement not only exceeding the meaningful clinical detectable change [34] but decreasing the risk of all-cause mortality as cutoff 10 reported by Pavasini et al. [39].

In addition, in physical performance and incorporated exercise, there are some noteworthy points of difference in comparing the previous studies conducted on the CCS service model [20,24]. First, our study could provide more validated results with a comparable study design and sample size calculation. Second, the intervention frequency and duration should be suitable for our participants to participate, with twice weekly for 3 months contrasting to Liang et al.’s once a week for 6 months and Chan et al.’s thrice weekly for 1 month [20,24]. Although it is difficult to consistently compare the effects among the 3 studies due to the different intervention durations and frequencies, these community older adults could still schedule their daily activity conveniently when leaving off the physical activity course. Furthermore, many studies have also indicated a structured group exercise held twice weekly and continuously for 3 months could benefit the physical performance of older adults [14,40]. Third, the accumulated timing (3 h weekly) and moderate intensity in the structured exercise design should meet the ACSM’s recommendations for community-dwelling older adults [29]. All of these could be adequate for our participants to improve their physical functions.

In the comparison group, we also found that physical performance levels were maintained among these older adults, even showing significant progress in FR performance. It seems that the program led by local trained community volunteers might have some benefits to community older adults, even without the structured group-based exercise toolkit program. Three major reasons we attempt to explain are as follows: First, the local community volunteers were trained on how to perform the activity program in the CCS. Second, the activity program given to the comparison group, though without a structured design, also included stretching and flexibility exercises and kicking or throwing differently sized balls, which is similar to the movement pattern of FR performance and promoted the participants’ balance ability. As a result, this reflected the improvement on FR performance. Following instructional training involving their peers, promoting community physical training programs for older adults became a feasible and successful model [42], even enhancing the maintenance of physical activity gains through a community-based intervention [43]. Therefore, the results could be attributed to the work of the local community volunteers with these participants, which warrants further investigations. Finally, all the participants had lived in the same community, and may have been more willing to participate in the program activities, shown by the increased attendance rate. These factors could have helped our participants improve and maintain their physical tests. Perhaps, the type of exercise toolkit could be educated to the local trained volunteer and applied in the CCS to approve if the similar effects occurred on physical function of the community older adults in the future.

Some limitations need to be mentioned in this study. First, we recruited our participants from the CCSs at a local area in a particular county, which could limit the generalizability of these effects to a broad population base. Second, we did not randomize our comparison group or design a “pure” control group without health-professional or peer-volunteer involvement. In fact, it is difficult and unethical to collect the CCS without any intervening program. Third, we only focused on investigation of physical function and not on the psychological and social aspects, and the long-term effects after the study ending remain unknown. These could be highlighted as future research directions. Fourth, we did not record the training volume or monitor the load of training on the participants in the two groups, which could bias our results. Finally, the CCS service model is so far generally implemented throughout Taiwan and connected to the long-term care service. However, due to limited resources of health professionals, we deem it would be valuable to assess the feasibility and investigate its effects of the inexpensive exercise toolkit run by peer volunteers among community-dwelling older adults participating in CCS services over a longer period. In doing so, we can fully understand the full value of the CCS service program incorporated with a structure exercise for aging-in-place, sustainability, and contributions to physical, mental and psychosocial health aspects in the long term.

## 5. Conclusions

In conclusion, the CCS service may be an effective strategy to respond to aging societies. Adding a structured group-based exercise program led by a PT using easily accessible and low-cost exercise tools into CCS services could promote most aspects of physical performance among community-dwelling older adults.

## Figures and Tables

**Table 1:** Name of all outcome tests, their objectives, skill evaluated or conditional capacity, and relevant clinical cutoff values.

Name of the Test	Objective	Skill Evaluated or Conditional Capacity	Relevant Clinical Cutoff Values
Short Physical Performance Battery (SPPB)	to evaluate lower extremity strength and physical mobility	Balance test: close feet, semi-tandem to tandem position without support Lower extremity strength: sit to stand X 5-time Gait ability: timed for walk 4 m	scores at or below 9 indicating mobility disability [34], all-cause mortality as cutoff 10 [39]
The one-leg stance (OLS) test	to assess postural steadiness while standing and screening for low functional level and frailty	to test one leg standing with flexing the opposite knee to allow the foot to clear the floor as long as possible	time < 5 s indicating more difficulty in transportation, high risk of functional dependency and frailty [35,36]
Functional reach (FR)	to evaluate the postural control and balance ability	the distance between the length of the middle-fingers’ tips and a maximal forward reach while maintaining 90° shoulder flexion and straight arms in the standing position	distance > 16 cm indicating low risk of fall and ADL disability [36]
The Timed Up and Go (TUG)	to determine the dynamic balance and fall risk	timed the individual rose from a chair, walked 3 m at a comfortable and safe pace, turned around, walked back to the chair, and sat down again	<13 s indicating high risk of fall; >16.5 s indicating high risk of ADL disability [36]
The 10 m walk test	functional mobility and walking ability	to record the time needed to walk to the mid-point of 10 m excluding the 5 m of each acceleration/deceleration phases	the Minimal Detectable Change values (MDC): 0.01–0.02 m/s; [38] cutoff for sarcopenia and frailty: 1 m/s [5,11]

**Table 2:** Comparison of the demographic data between intervention and control groups.

Variables	Intervention Group (*n* = 42)	Control Group (*n* = 41)	*p* Value
Age (y)	76.9 ± 7.3	77 ± 6.3	0.95
Height (cm)	153.5 ± 7.9	153.0 ± 7.8	0.78
Weight (kg)	59.3 ± 12.4	58.0 ± 9.8	0.61
Body mass index	25.0 ± 4.0	24.7 ± 2.9	0.68
Gender			0.85
Female	11 (26)	10 (24)	
Male	31 (74)	31 (76)	
Education			0.1
Illiterate	21 (50)	12 (29)	
Elementary school	17 (40)	20 (49)	
Junior height school and above	4 (10)	9 (22)	
Chronic disease			
Hypertension	23 (55)	22 (54)	0.92
Diabetes	14 (33)	12 (29)	0.69
Heart disease	10 (24)	12 (29)	0.57
Arthritis	9 (21)	7 (17)	0.62
Stroke	4 (10)	0 (0)	0.12

Continuous values are presented as means ± standard deviations. Categorical variables are presented as *n* (%).

**Table 3:** Comparison of the physical performance tests between intervention and control groups.

Variables	Intervention Group (*n* = 42)				Control Group (*n* = 41)				Main Effect (Group)		Main Effect (Time)		Interaction (Group × Time)	
	Pre-Test		Post-Test		Pre-Test		Post-Test							
	Mean	(SD)	Mean	(SD)	Mean	(SD)	Mean	(SD)	F	*p*	F	*p*	F	*p*
SPPB	10.0	(1.9)	11.3	(1.4)	9.7	(2.3)	9.9	(2.5)	3.60	0.06	26.24	<0.01	12.35	<0.01
One leg stance (s)	6.0	(5.4)	12.5	(8.6)	9.5	(10.6)	9.8	(10.7)	0.05	0.83	40.09	<0.01	33.49	<0.01
Forward reach (cm)	14.8	(5.3)	19.9	(4.5)	15.0	(6.7)	16.5	(5.2)	2.23	0.14	48.22	<0.01	14.50	<0.01
TUG (s)	11.1	(3.3)	9.6	(3.1)	11.8	(4.5)	11.3	(4.5)	2.16	0.15	23.24	<0.01	6.15	0.02
10-m WT (s)	10.6	(2.9)	8.9	(2.3)	11.0	(3.9)	10.6	(4.1)	2.25	0.14	31.43	<0.01	13.28	<0.01

Abbreviations: SPPB, Short Physical Performance Battery. TUG, Timed up and go test, 10-m WT, 10 m walking time.

## Data Availability

The datasets generated and analyzed during this study are available from the corresponding author on reasonable request.
